# Pott's Spine Unveiled: A Comprehensive Case Report and Surgical Intervention

**DOI:** 10.7759/cureus.60028

**Published:** 2024-05-10

**Authors:** Sachin Goel, Sohael Khan, Kashyap Kanani, Suhit Naseri, Vivek H Jadawala, Anmol Suneja

**Affiliations:** 1 Orthopaedics, Jawaharlal Nehru Medical College, Datta Meghe Institute of Higher Education and Research, Wardha, IND; 2 Pathology, Jawaharlal Nehru Medical College, Datta Meghe Institute of Higher Education and Research, Wardha, IND

**Keywords:** neurological deficits, tuberculosis spondylitis, corpectomy, anterior cervical decompression, discitis, vertebral osteomyelitis

## Abstract

This case report describes the presentation, diagnosis, and surgical management of a 61-year-old female admitted to a tertiary care hospital with a two-month history of neck pain and weakness in all four limbs. Despite the absence of a clear history of trauma, a detailed examination revealed restricted neck flexion, paraspinal muscle spasm, and neurological deficits. Contrast-enhanced MRI indicated vertebral osteomyelitis and discitis at the C5-C6 level, with a suspected infective etiology, possibly tuberculosis spondylitis. The patient underwent anterior cervical decompression, corpectomy of C5-C6, and fusion of C4-C7. Postoperative management included intravenous antibiotics, physiotherapy, and anti-tubercular treatment. The patient exhibited satisfactory recovery, and this case underscores the importance of comprehensive evaluation and prompt intervention in managing complex spinal infections.

## Introduction

Spinal infections, including vertebral osteomyelitis and discitis, pose significant challenges in diagnosis and management due to their diverse etiologies and variable clinical presentations [1]. Pott's spine, a form of tuberculous spondylitis, represents a rare but severe manifestation of spinal infection that requires prompt intervention for optimal outcomes [2]. The insidious onset, absence of overt trauma, and nonspecific symptoms often contribute to delayed diagnosis and treatment initiation [3]. Tuberculosis remains a primary global health concern, with extrapulmonary manifestations, such as spinal involvement, accounting for a substantial burden of morbidity and disability [4]. In particular, the cervical spine is a relatively uncommon but critical site for tuberculous spondylitis, necessitating a high index of suspicion for timely diagnosis [5].

Advancements in imaging modalities, such as magnetic resonance imaging (MRI), have revolutionized the diagnostic landscape, enabling clinicians to delineate the extent and nature of spinal infections with greater precision [6]. The role of surgery in managing spinal infections has evolved, with anterior cervical decompression and corpectomy emerging as effective interventions in selected cases [2]. Given the complexity and varied presentations of spinal infections, there is a need for comprehensive case reports that document the clinical journey, diagnostic challenges, and therapeutic strategies employed in individual patients. This case report contributes to the existing literature by presenting a detailed account of a patient with Pott's spine managed with anterior cervical decompression and corpectomy.

## Case presentation

**Figure 1:**
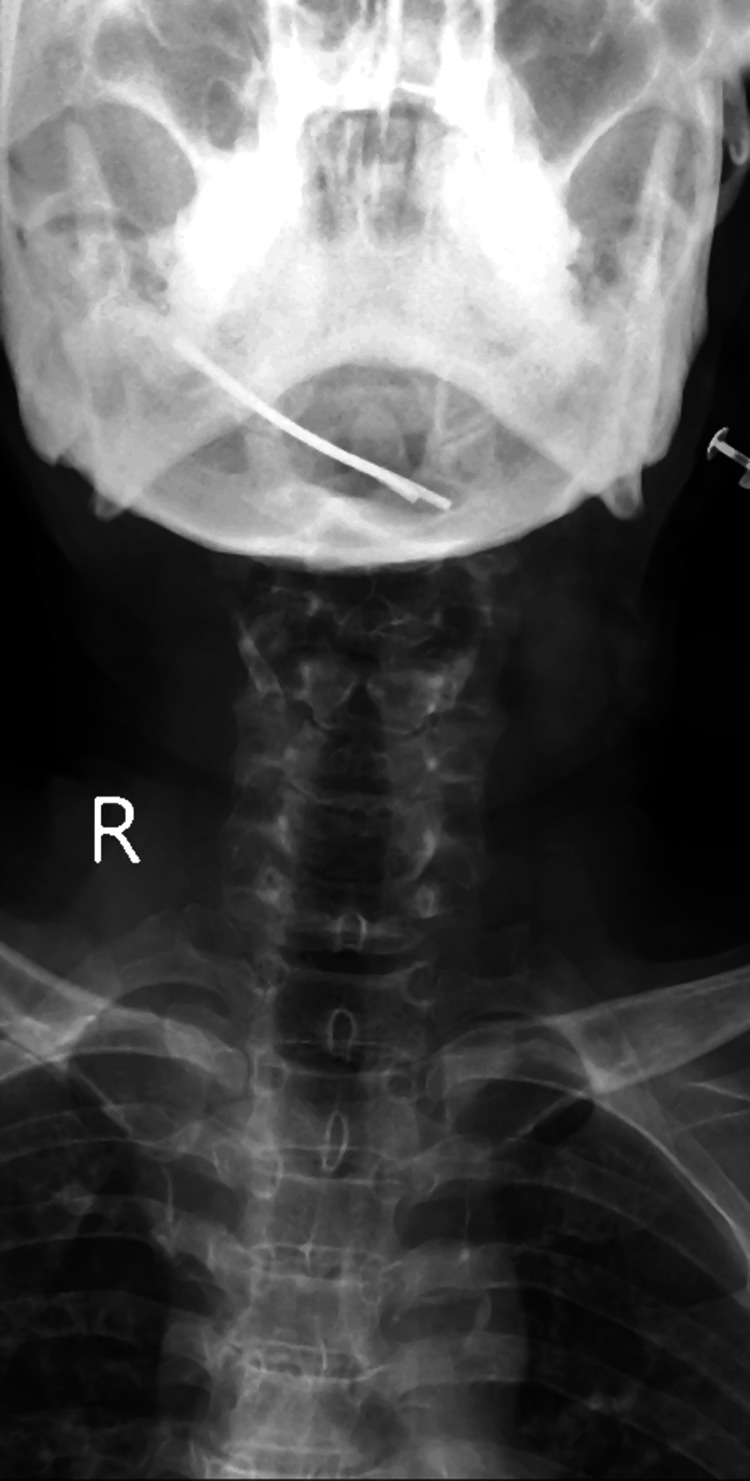
Preoperative image of the patient

A 61-year-old woman was brought to the emergency department of a tertiary care hospital with complaints of neck pain and weakness in all four limbs, which had persisted for the past two months. Although there was no apparent history of accident or trauma, the patient's relatives disclosed that she had been unable to walk during this period. Upon inspection, a comprehensive physical examination in supine and sitting positions revealed normal cervical spine and spinal curvature. However, tenderness was observed on palpation of the cervical spine, accompanied by paraspinal muscle spasm. Neck flexion was restricted and terminally painful, with a complete absence of finger grip and active finger movements on both sides. Bony tenderness and paraspinal muscle spasm were noted on palpation of the L5 spine. A radiological examination is shown in Figure 1.

**Figure 2:**
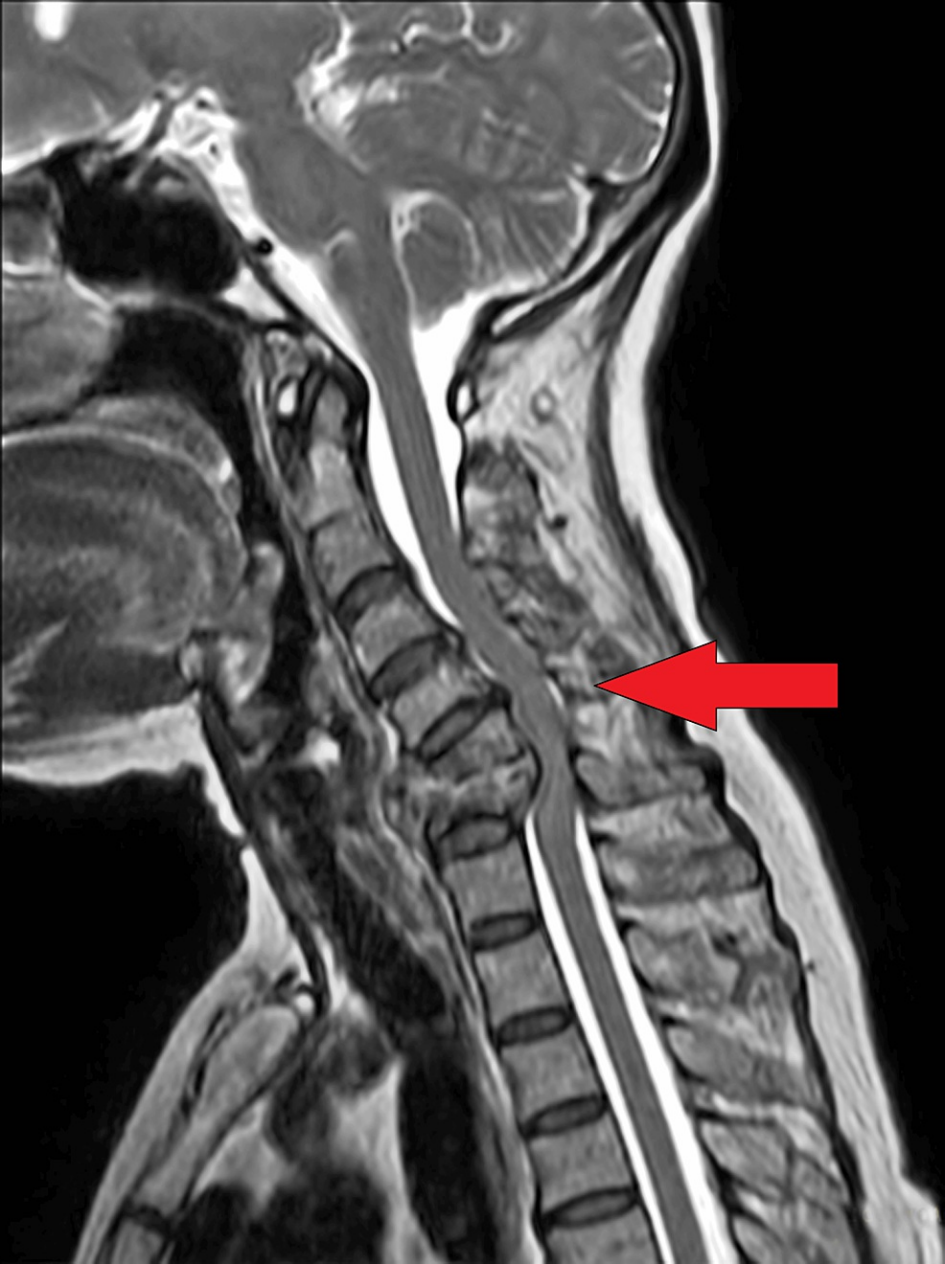
C5 and C6 vertebrae (vertebral osteomyelitis) and C5-C6 disc levels (discitis) with subligamentous extensions

The patient was referred to the inpatient department following a thorough history collection and physical examination. Blood tests and MRI of the spine were recommended in the medicine department. The contrast-enhanced MRI revealed evidence of a hemangioma at the C7 vertebra and an enhancing altered signal intensity lesion involving C5 and C6 vertebrae (vertebral osteomyelitis) and C5-C6 disc levels (discitis) with subligamentous extensions (Figure 2). The findings suggested a likely infective etiology, possibly tuberculosis spondylitis at the C5-C6 level. Consequently, the physician diagnosed the patient with Pott's spine affecting the C5 and C6 vertebrae, and the patient was transferred to the neurological department for further surgical management.

**Figure 3:**
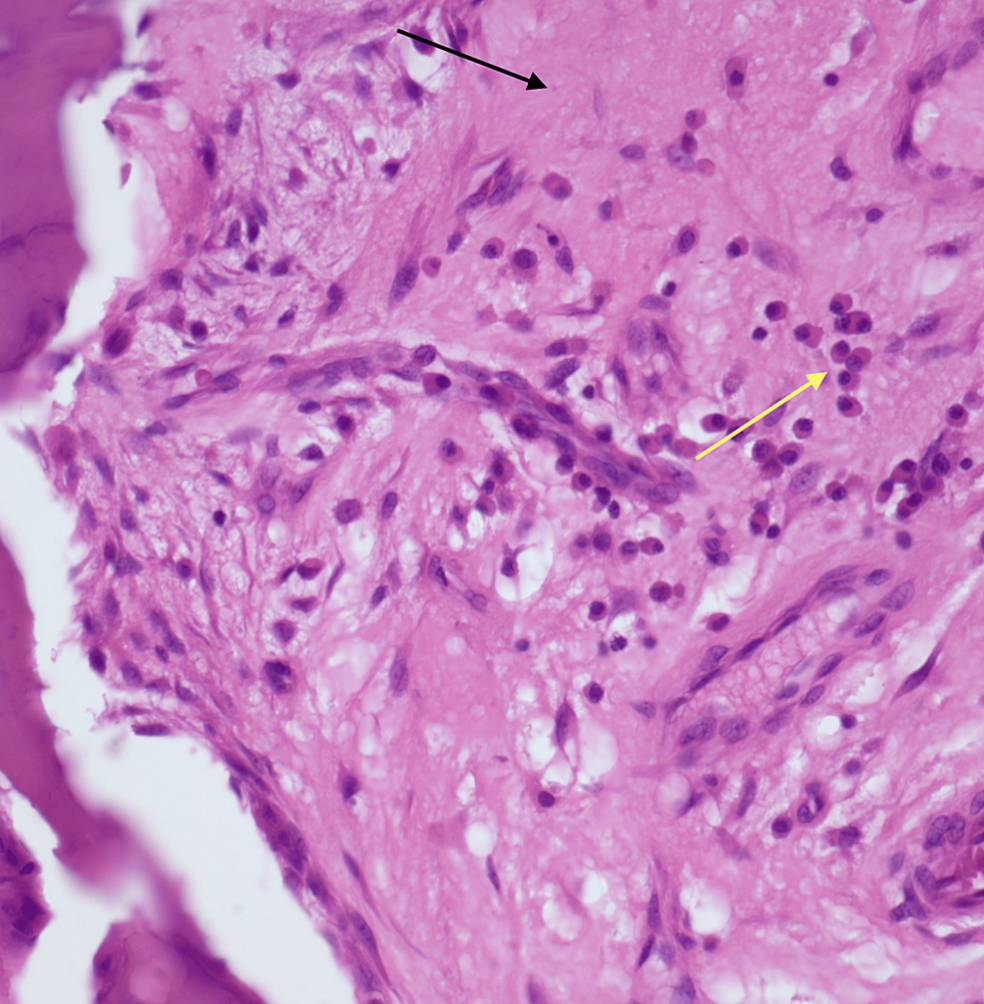
Section from the bony chronic inflammation with bone destruction. Marrow is replaced by fibrosis (black arrow) with plasma cells (yellow arrow) and lymphocytes (H&E, 40×)

Upon admission to the neurological department, the neurosurgeon reviewed all the patient's investigations and planned surgery. The patient underwent anterior cervical decompression and corpectomy of the C5-C6 level, as well as a fusion of the C4-C7 levels under general anesthesia. The surgical procedure involved a 2-cm incision over the left iliac crest for bone graft preparation, followed by a 4-cm longitudinal incision over the left sternocleidomastoid for exposure and identification of the vertebral bodies from C4 to C7, confirmed under C-arm guidance. Decompression and corpectomy at C5-C6 were performed, and a four-hole expandable plate was inserted on C4 and C7 processes, secured with two locking screws of 4 mm × 16 placed proximally and distally to the plate. The harvested bone graft was inserted at C5 and C6, followed by thorough irrigation with normal saline and sterile dressing. Histopathology revealed evidence of bony chronic inflammation with associated bone destruction. Marrow is replaced by fibrosis (black arrow) with plasma cells (yellow arrow) and lymphocytes (Figure 3). 

**Figure 4:**
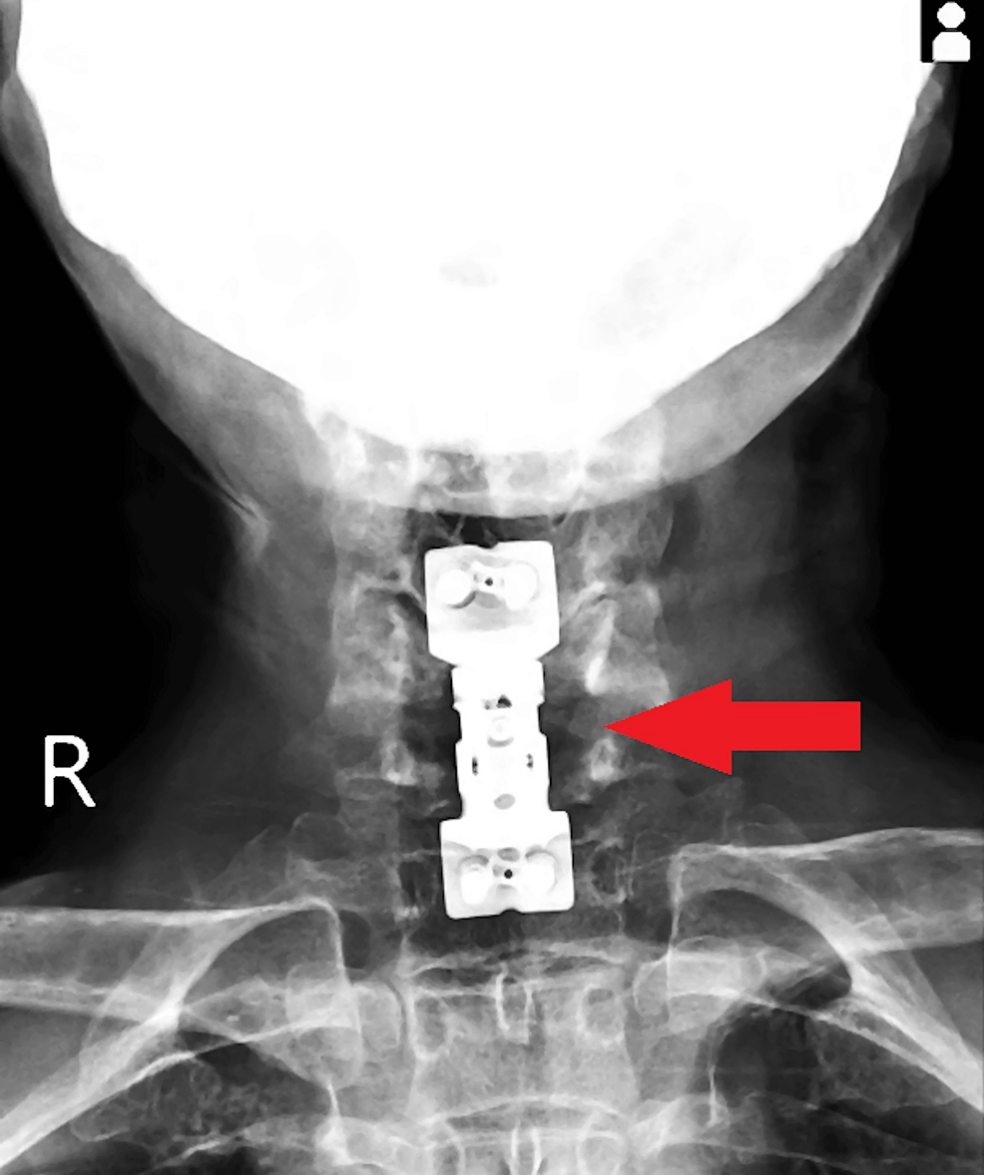
Postoperative X-ray of the patient

Postoperatively, the patient was managed with intravenous antibiotics. On the second day after surgery, sterile dressing was performed, sutures were intact, and an X-ray confirmed satisfactory results (Figure 4). Physiotherapy, including ankle pumps, passive joint mobilization, bedside sitting, and wheelchair mobilization, was initiated on the second postoperative day. On the third day, anti-tubercular treatment was commenced. Repeated sterile dressing on the seventh day revealed a healthy and intact suture site. Total weight-bearing mobilization with a walker started on the 11th day, and sutures were removed on the 12th day, with a healthy wound site. The patient's vital signs were stable, general condition was fair, cervical hard collar remained in situ, and there was active range of motion in fingers, wrists, knees, ankles, and toes, as well as intact distal sensation and circulation. Consequently, the doctor planned for the patient's discharge.

## Discussion

This case report highlights the complexity and successful management of Pott's spine, a form of tuberculous spondylitis, through anterior cervical decompression and corpectomy. The patient's presentation with insidious neck pain, weakness in all four limbs, and the absence of overt trauma underscores the challenging nature of diagnosing spinal infections, necessitating a comprehensive approach [6]. The diagnostic journey in this case was facilitated by advanced imaging techniques, particularly contrast-enhanced MRI, which revealed characteristic findings of vertebral osteomyelitis and discitis at the C5-C6 level [7]. Identifying a hemangioma at the C7 vertebra added a layer of complexity to the diagnostic process, emphasizing the importance of thorough imaging interpretation [8]. The clinical suspicion of an infective etiology, possibly tuberculosis spondylitis, aligned with global trends, as extrapulmonary manifestations of tuberculosis, including spinal involvement, continue to pose diagnostic challenges [4,9].

Surgical intervention in the form of anterior cervical decompression and corpectomy proved effective in addressing the patient's neurological deficits and achieving successful fusion of the affected vertebrae [10]. This aligns with contemporary literature advocating for surgery in selected cases of spinal infections to prevent neurological deterioration and achieve spinal stability [1]. An expandable plate and cortico-cancellous bone graft from the left iliac crest demonstrated a multidimensional approach to decompression, stability, and fusion [2].

Postoperative management, including intravenous antibiotics, physiotherapy, and anti-tubercular treatment, played a crucial role in the patient's recovery. The timely initiation of anti-tubercular treatment on the third postoperative day aimed to target the infective etiology and prevent further complications [2]. The successful outcome in this case, evidenced by the patient's improved neurological status, absence of complications, and the ability to resume weight-bearing mobilization, supports the effectiveness of the chosen surgical and medical interventions [7]. While the case report provides valuable insights into managing Pott's spine, it is essential to acknowledge certain limitations. The rarity of this condition may limit the generalizability of findings, and individual variations in patient response to treatment must be considered.

## Conclusions

The presented case of Pott's spine underscores the intricate diagnostic challenges and successful management strategies associated with tuberculous spondylitis. The patient's clinical presentation, marked by insidious neck pain, limb weakness, and absence of overt trauma, emphasizes the nuanced nature of spinal infections that often elude immediate recognition. Utilizing advanced imaging techniques, particularly contrast-enhanced MRI, played a pivotal role in delineating the extent of vertebral osteomyelitis and discitis. The chosen surgical intervention, anterior cervical decompression, and corpectomy, demonstrated efficacy in alleviating neurological deficits and achieving spinal stability. Postoperative care, including intravenous antibiotics, physiotherapy, and early initiation of anti-tubercular treatment, contributed to the favorable outcome observed in the patient's improved neurological status and successful postoperative recovery. While this case adds valuable insights to the literature on spinal infections, the rarity of Pott's spine underscores the need for continued research and collaborative efforts to refine diagnostic and therapeutic approaches for optimal patient outcomes in similar complex clinical scenarios.

## References

[1] Gouliouris T, Aliyu SH, Brown NM (2010). Spondylodiscitis: update on diagnosis and management. J Antimicrob Chemother.

[2] Tuli SM (2007). Tuberculosis of the spine: a historical review. Clin Orthop Relat Res.

[3] Moon MS (1997). Tuberculosis of the spine. Controversies and a new challenge. Spine (Phila Pa 1976).

[4] (2023). Global tuberculosis report 2020. https://www.who.int/publications-detail-redirect/9789240013131.

[5] Rajasekaran S (2001). The natural history of post-tubercular kyphosis in children. Radiological signs which predict late increase in deformity. J Bone Joint Surg Br.

[6] Garg RK, Somvanshi DS (2011). Spinal tuberculosis: a review. J Spinal Cord Med.

[7] Rauf F, Chaudhry UR, Atif M, ur Rahaman M (2015). Spinal tuberculosis: our experience and a review of imaging methods. Neuroradiol J.

[8] Jain AK (2016). Tuberculosis of spine: research evidence to treatment guidelines. Indian J Orthop.

[9] Rajasekaran S, Soundararajan DC, Shetty AP, Kanna RM (2018). Spinal tuberculosis: current concepts. Global Spine J.

[10] Rajasekaran S (2013). Natural history of Pott's kyphosis. Eur Spine J.

